# Identifying novel acute pancreatitis sub-phenotypes using total serum calcium trajectories

**DOI:** 10.1186/s12876-024-03224-9

**Published:** 2024-04-23

**Authors:** Chang-li Li, Xing-chen Lin, Meng Jiang

**Affiliations:** 1 Department of FSTC Clinic, The First Affiliated Hospital, Zhejiang University School of Medicine, 310003 Hangzhou, China; 2 Emergency and Trauma Center, The First Affiliated Hospital, Zhejiang University School of Medicine, #79 Qingchun Road, Hangzhou 310003, Zhejiang Province PR China

**Keywords:** Acute pancreatitis, Total serum calcium, Group-based trajectory modeling, Sub-phenotypes

## Abstract

**Background:**

Acute pancreatitis (AP) has heterogeneous clinical features, and identifying clinically relevant sub-phenotypes is useful. We aimed to identify novel sub-phenotypes in hospitalized AP patients using longitudinal total serum calcium (TSC) trajectories.

**Methods:**

AP patients had at least two TSC measurements during the first 24 h of hospitalization in the US-based critical care database (Medical Information Mart for Intensive Care-III (MIMIC-III) and MIMIC-IV were included. Group-based trajectory modeling was used to identify calcium trajectory phenotypes, and patient characteristics and treatment outcomes were compared between the phenotypes.

**Results:**

A total of 4518 admissions were included in the analysis. Four TSC trajectory groups were identified: “Very low TSC, slow resolvers” (*n* = 65; 1.4% of the cohort); “Moderately low TSC” (*n* = 559; 12.4%); “Stable normal-calcium” (*n* = 3875; 85.8%); and “Fluctuating high TSC” (*n* = 19; 0.4%). The “Very low TSC, slow resolvers” had the lowest initial, maximum, minimum, and mean TSC, and highest SOFA score, creatinine and glucose level. In contrast, the “Stable normal-calcium” had the fewest ICU admission, antibiotic use, intubation and renal replace treatment. In adjusted analysis, significantly higher in-hospital mortality was noted among “Very low TSC, slow resolvers” (odds ratio [OR], 7.2; 95% CI, 3.7 to 14.0), “moderately low TSC” (OR, 5.0; 95% CI, 3.8 to 6.7), and “Fluctuating high TSC” (OR, 5.6; 95% CI, 1.5 to 20.6) compared with the “Stable normal-calcium” group.

**Conclusions:**

We identified four novel sub-phenotypes of patients with AP, with significant variability in clinical outcomes. Not only the absolute TSC levels but also their trajectories were significantly associated with in-hospital mortality.

**Supplementary Information:**

The online version contains supplementary material available at 10.1186/s12876-024-03224-9.

## Introduction

Acute pancreatitis (AP) is one of most common gastrointestinal diseases that requiring acute hospital admission [1], with about 2,814,972 incident cases occurred each year in the globe [2]. Most patients present with mild AP, which is self-limiting and often resolves within one week. Approximately 1/5 of patients develop moderate or severe AP, with necrosis of the pancreatic tissue and extrapancreatic organ dysfunction, and a substantial mortality rate of 20–40% [3–6].

According to the revised Atlanta classification, the severity of AP can be defined as mild, moderately severe, or severe [7]. Given the unpredictable course of AP, a plethora of clinical and biochemical scoring systems have been developed to predict the prognosis of AP, such as Acute Physiology and Chronic Health Evaluation II and Ranson’s score [8, 9]. These scoring systems are based on a combination of vital signs, certain laboratory data, and radiographic findings, which are frequently cumbersome to calculate and thus limited their application in clinical practice [9, 10].

Several individual serum tests have been studied as predictors of severe AP, including C-reactive protein (CRP), blood urea nitrogen (BUN), procalcitonin, and others [10, 11]. Among them, high level of CRP was found to be the most promising marker that correlated with pancreatic necrosis and severe AP course [12]. However, CRP levels may be influenced by liver disease [13], and its levels peak at 72–96 h after symptom onset, which may limit its prognostic accuracy as an early indicator [14, 15].

Hypocalcemia is one of the most common electrolyte abnormalities among AP [16, 17]. It’s proposed that hypocalcemia might result from fat necrosis and the subsequent “calcium soaps” formation [18]. Current research has revealed that the in-depth reason for the reduction in serum calcium is the suppression of cytomembrane Ca2 + ATP enzyme at the outbreak of AP, which results in an influx of ectocytic calcium ions and intracellular calcium overload [19]. Since intracellular calcium overload is the central mechanism of acinar cell injury in AP [19], theoretically low serum calcium level can reflect both the mechanism of pancreatic injury, and the consequence of peripancreatic fat tissue necrosis. In hospitalized patients, serum calcium is measured routinely and repeatedly, and each patient’s calcium trajectory provides a repository of longitudinal quantitative data. There is a paucity of report on using this longitudinal data for phenotyping and prognosticating patients with AP.

The aim of this study was to classify AP into novel sub-phenotypes based on their calcium trajectories. We hypothesized that each of these trajectory groups would have different physiological characteristics and clinical outcomes. Looking at calcium trends in addition to a point-specific calcium level might better predict prognosis.

## Methods

### Source of data

We conducted this retrospective study based on the sizeable critical care database of Medical Information Mart for Intensive Care (MIMIC)-IV (version 1.0) [20]. The MIMIC-IV database is an integrated and comprehensive clinical dataset containing all the patients admitted to the ICUs of Beth Israel Deaconess Medical Center in Boston, MA, from 2008 to 2019. There were 524,520 distinct hospital admissions for 257,366 adult patients (aged 16 years or above) during the study period. Besides, we also searched for eligible patients from the old version of MIMIC-III between 2001 and 2007. One author (M.J) who obtained access to this database was responsible for data extraction. Since the study was an analysis of a third-party anonymized publicly available database, institutional review board (IRB) approval from our institution was exempted.

### Participants

Patients with AP (ICD-9: 5770) were identified using the revised Atlanta definition [7], which is based on the fulfilment of two of three features: (1) abdominal pain, (2) serum amylase (or lipase) of at least three times the upper limit of normal, or (3) findings consistent with AP on contrast-enhanced CT, MRI, or transabdominal ultrasound. In MIMIC database, total serum calcium (TSC) was mostly measured, thus in this study TSC was used for analysis. Only TSC measurements from Hour 0 (defined as time of admission) to Hour 24 were included. The TSC data from Hour 0 to Hour 24 were split into 1-hour blocks of time. If a patient had multiple TSC measures in that 1-hour block, the earliest TSC measurement was used. Patients who had at least two TSC measurements during 0–24 h were included.

### Outcome definition

In-hospital mortality was the primary outcome. Other clinical outcomes such as ICU admission, length of hospitalization, antibiotic use, intubation rate, and renal replace treatment (RRT) were secondary outcomes.

### Management of missing data

In this study, the continuous variables CRP and lactate had more than 50% of values missing, so they were excluded from the analysis. Most of the other continuous variables (e.g., albumin, bicarbonate, BUN) had less than 5% of values missing, the missing values were handled with single imputation in the analysis [21].

### Sensitivity analysis

To test the robustness of our findings, we also constructed trajectory models by excluding patients who died or were transferred before Hour 24, using 4-hour time block of TSC measurement, based on the TSC measurements in patients with calcium infusion during the first 24 h after admission, as well as using available serum ionized calcium. The consistency of the discriminability of these models was investigated.

### Statistical analysis

Categorical variables are presented as number and proportions, continuous variables are summarized as the mean ± standard deviation (SD) or the median (interquark range [IQR]) according to the data distribution. Demographics, comorbidities, laboratory results, and processes of care (e.g., intubation, RRT) were compared between survivors and non-survivors. In addition, TSC measurements (initial, mean, maximum, minimum, and TSC variability, defined as the SD of TSC measurements during the first 24 h) were also compared between survivors and non-survivors. For these comparisons, categorical and continuous variables were tested for statistical significance using Chi-square tests, Fisher’s exact tests, or Wilcoxon rank-sum tests, as appropriate.

We used group-based trajectory modeling to identify sub-phenotypes based on TSC trajectories from the first 24 h of TSC data. Group-based trajectory modeling is a specialized mixture modeling method that is used to identify groups of individuals following similar trajectories for a particular variable [22]. The Bayesian information criterion and average posterior probability were used for model selection, which ensure that a well-fitting model is chosen. We explored two-, three-, and four-group trajectories, with both linear and quadratic models investigated. Group-based trajectory modeling was conducted using the ‘traj’ package in Stata software (StataCorp).

Demographics, comorbidities, physiological characteristics, and clinical outcomes were compared between the identified trajectory groups with analysis of Chi-square tests and variance rank tests. Multivariable logistic regression analysis was performed to evaluate the independent association between TSC trajectories and in-hospital mortality (controlling for age, sex, race and comorbidities), using “stable normal-calcium” as the reference group. Two-tailed P values < 0.05 were considered statistically significant for all tests, and analyses were performed using Stata version 16.0 software.

## Results

### Patient characteristics

**Fig. 1:**
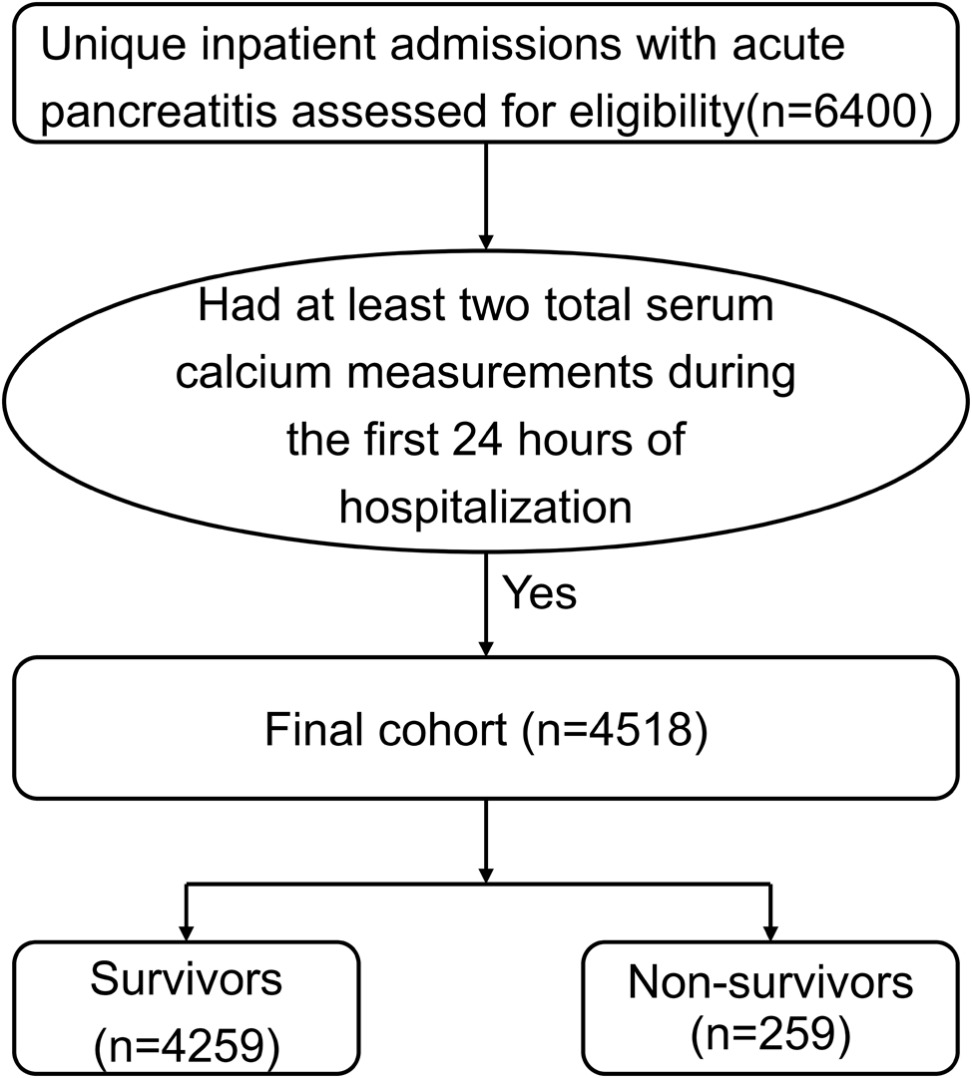
Diagram of patient selection for the study population

**Fig. 2:**
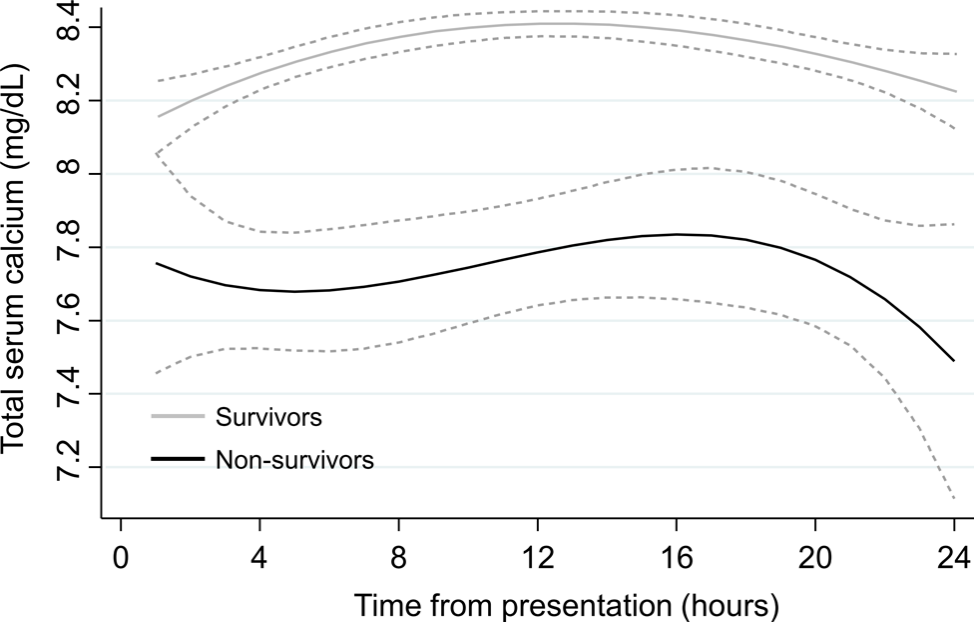
Total serum calcium (TSC) over time in survivors and non-survivors. Grey dotted line represents the 95% confidence interval of TSC measurements

Of the 6400 patients with AP, 4518 had at least two TSC measurements during the first 24 h of hospitalization (Fig. 1). There were 4259 survivors and 259 non-survivors, with an overall in-hospital mortality rate of 5.7% (95% CI: 5.1-6.5%) and median length of stay of 5.4 days (IQR, 3.0–10.6) in hospital. The median time to death for non-survivors was 13.1 days (IQR, 4.1–25.5) after admission. As shown in Table S1, the maximum amylase and lipase didn’t differ significantly between the survivors and non-survivors. However, the non-survivors had a significantly higher SOFA score on admission, and were more likely to be admitted to ICU, receive antibiotic use, undergone intubation and RRT. Non-survivors were older than survivors, with a mean age of 70.1 ± 35.8 years compared with 56.9 ± 24.5 years. Non-survivors had higher rates of congestive heart failure, chronic renal failure, metastatic cancer, and liver disease. Initial TSC was lower in the non-survivors than in survivors (7.8 ± 1.2 mg/dL vs. 8.5 ± 0.9 mg/dL; *P* < 0.001), as were the mean, maximum, and minimum TSC. Furthermore, TSC variability, as assessed by the SD of TSC measurements, was higher in non-survivors than in survivors (*P* < 0.001). The survivors and non-survivors followed distinct TSC trajectories: survivors had a slight low initial TSC followed by a gradual increase in TSC, whereas non-survivors had a significantly lower initial TSC followed by a fluctuating low level of TSC (Fig. 2).

### Group-based TSC trajectory modeling

**Fig. 3:**
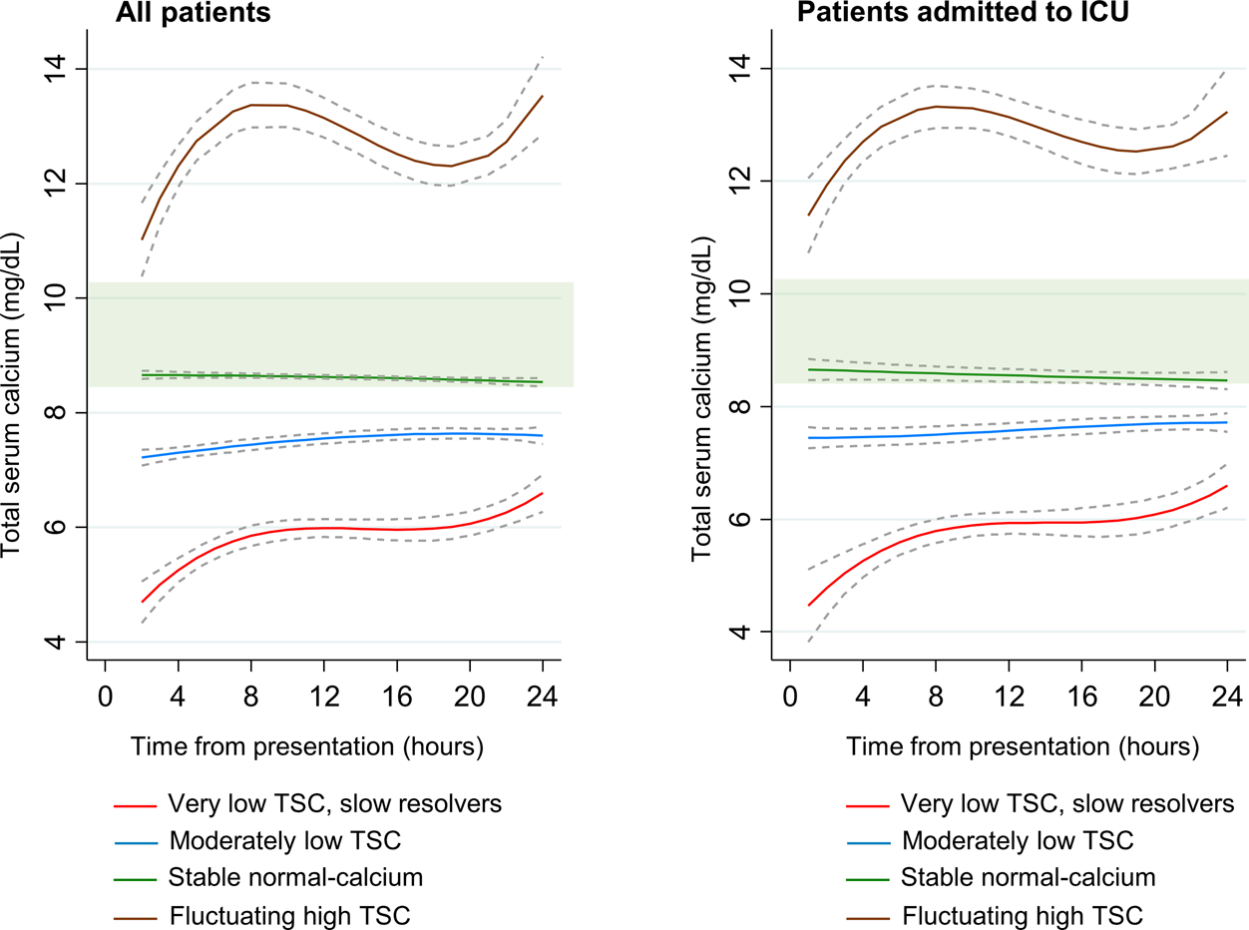
Total serum calcium (TSC) phenotypes in acute pancreatitis (AP) patients. Using group-based trajectory modeling, four TSC trajectory group sub-phenotypes were identified: “Very low TSC, slow resolvers”; “Moderately low TSC”; “Stable normal-calcium”; and “Fluctuating high TSC”

The sub-phenotype selection process of the trajectory model is shown in Table S2. Analysis of the different group-based trajectory models revealed that the four- phenotype quadratic function model had the optimal fit (Fig. 3): Phenotype 1 (very low TSC, slow resolvers, *n* = 65); Phenotype 2 (Moderately low TSC, *n* = 559); Phenotype 3 (Stable normal-calcium, *n* = 3875); and Phenotype 4 (Fluctuating high TSC, *n* = 19). Each of the four TSC trajectories was found to be the product of a unique quadratic function describing TSC as a function of time. TSC trajectories were also modeled separately in patients admitted to the ICU, and the overall trajectories remained stable (Fig. 3).

In a sensitivity analysis to evaluate for informative dropout in the cohort, patients who died or were transferred before Hour 24 were excluded, and a new trajectory model was developed. This new model classified 96% of the patients into the same phenotypes as the original model. Similarly, in another sensitivity analysis to account for frequency of TSC measurements, a 4-hour time block (instead of a 1-h time block) model was explored, and this model classified 93% of the patients into the same phenotypes as the original model (data not shown).

### TSC characteristics within phenotypes

Most of the patients with “Stable normal-calcium” phenotype had normal TSC records, and only 31.3% of these patients had transient hypocalcemia (TSC < 8.4 mg/dL) in the first 24 h (Table 1). “Very low TSC, slow resolvers” had the lowest initial, maximum, minimum, and mean TSC, and highest SOFA score, creatinine and glucose level (Table 2). In-hospital mortality was highest in the “Very low TSC, slow resolvers” group (21.5%), followed by the “Moderately low TSC” and “Fluctuating high TSC” (17.2% and 15.8%, respectively), with the “Stable normal-calcium” having the lowest mortality (3.8%).

**Table 1:** Comparison of total serum calcium within the four calcium-trajectory phenotypes

	Very low TSC, slow resolvers (*n* = 65)	Moderately low TSC (*n* = 559)	Stable normal-calcium (*n* = 3875)	Fluctuating high TSC (*n* = 19)	*P*
Initial TSC, mg/dL	5.4 ± 1.1	7.2 ± 0.6	8.6 ± 0.6	12.4 ± 1.9	< 0.001
Maximum TSC, mg/dL	6.4 ± 0.9	7.6 ± 0.6	8.7 ± 0.6	13.1 ± 1.7	< 0.001
Minimum TSC, mg/dL	5.1 ± 1.0	6.9 ± 0.6	8.6 ± 0.6	11.6 ± 1.6	< 0.001
Mean TSC, mg/dL	5.8 ± 0.7	7.3 ± 0.4	8.6 ± 0.5	12.3 ± 1.3	< 0.001
TSC, SD	0.9 ± 0.5	0.5 ± 0.4	0.3 ± 0.2	1.2 ± 0.8	< 0.001
Time to trough TSC, h	9.1 ± 6.6	10.6 ± 6.9	12.5 ± 5.7	11.4 ± 7.0	< 0.001
Hypocalcaemia (TSC < 8.4 mg/dL), n (%)	65 (100.0)	548 (98.0)	1213 (31.3)	0 (0.0)	< 0.001

TSC, total serum calcium; SD, standard deviation

**Table 2:** Comparison of patient characteristics, therapy, and outcome within the four calcium-trajectory phenotypes

	Very low TSC, slow resolvers (*n* = 65)	Moderately low TSC (*n* = 559)	Stable normal-calcium (*n* = 3875)	Fluctuating high TSC (*n* = 19)	*P*
Age (years)	54.3 ± 15.0	57.6 ± 17.4	56.3 ± 17.0	55.0 ± 13.4	0.232
Male, n (%)	36 (55.4)	298 (53.3)	2012 (51.9)	13 (68.4)	0.447
Race, n (%)					< 0.001
White	50 (76.9)	343 (61.4)	2672 (69.0)	13 (68.4)	
Black	2 (3.1)	58 (10.4)	533 (13.8)	1 (5.3)	
Other	13 (20.0)	158 (28.3)	670 (17.3)	5 (26.3)	
Comorbidities, n (%)					
Congestive heart failure	9 (13.8)	88 (15.7)	411 (10.6)	1 (5.3)	0.003
Chronic pulmonary disease	13 (20.0)	91 (16.3)	632 (16.3)	2 (10.5)	0.774
Chronic renal failure	11 (16.9)	69 (12.3)	454 (11.7)	5 (26.3)	0.136
Metastatic cancer	3 (4.6)	8 (1.4)	113 (2.9)	2 (10.5)	0.029
Liver disease	23 (35.4)	173 (30.9)	692 (17.9)	4 (21.1)	< 0.001
Disease severity and laboratory indexes					
SOFA score (median [IQR])	8.0 [4.0, 13.0]	6.0 [3.0, 10.0]	4.0 [2.0, 7.0]	5.0 [3.0, 10.0]	< 0.001
Minimum albumin, mg/dL	2.5 ± 0.6	2.8 ± 0.6	3.4 ± 0.7	2.9 ± 0.4	< 0.001
Minimum bicarbonate, mmol/L	15.8 ± 4.8	18.4 ± 5.3	24.0 ± 4.2	23.1 ± 6.7	< 0.001
Maximum bilirubin, mg/dL	2.0 ± 2.1	2.6 ± 4.2	1.9 ± 3.5	2.4 ± 2.5	0.004
Maximum creatinine, mg/dL	2.6 ± 2.2	2.0 ± 2.4	1.2 ± 1.3	2.2 ± 1.9	< 0.001
Maximum BUN, mg/dL	35.7 ± 27.4	30.7 ± 26.8	18.6 ± 17.9	42.3 ± 26.2	< 0.001
Maximum glucose, mg/dL	225.2 ± 125.8	185.9 ± 118.1	132.2 ± 83.1	162.4 ± 88.7	< 0.001
Maximum white blood cell count (10^9/L)	14.4 ± 8.5	14.8 ± 8.5	10.6 ± 7.4	12.0 ± 5.3	< 0.001
Maximum amylase, IU/L	383.0 [118.0, 702.0]	212.0 [77.0, 580.0]	144.0 [64.0, 484.0]	54.0 [43.0, 185.5]	0.001
Maximum lipase, IU/L	617.0 [230.8, 1225.2]	292.0 [78.0, 920.0]	156.0 [49.0, 714.5]	96.0 [37.0, 209.0]	< 0.001
Clinical outcomes					
In-hospital death	14 (21.5)	96 (17.2)	146 (3.8)	3 (15.8)	< 0.001
ICU admission, n (%)	58 (89.2)	470 (84.1)	1110 (28.6)	16 (84.2)	< 0.001
Hospital length of stay, days, (median [IQR])	20.6 [11.8, 32.9]	10.0 [4.9, 18.1]	4.9 [2.8, 9.0]	16.6 [8.3, 23.7]	< 0.001
Antibiotic use, n (%)	55 (84.6)	364 (65.1)	1719 (44.4)	13 (68.4)	< 0.001
Intubation, n (%)	38 (58.5)	234 (41.9)	346 (8.9)	7 (36.8)	< 0.001
RRT, n (%)	20 (30.8)	49 (8.8)	71 (1.8)	3 (15.8)	< 0.001

SOFA, Sequential Organ Failure Assessment; BUN, Blood urea nitrogen; RRT, renal replace treatment

After adjusting for potential confounders in model 3 of Table 3, significantly higher odds of in-hospital mortality was noted among “Very low TSC, slow resolvers” (odds ratio [OR], 7.2; 95% CI, 3.7 to 14.0), “moderately low TSC” (OR, 5.0; 95% CI, 3.8 to 6.7), and “Fluctuating high TSC” (OR, 5.6; 95% CI, 1.5 to 20.6) groups compared with the “Stable normal-calcium” group.

**Table 3:** Association between in-hospital death and calcium-trajectory phenotype in logistic regression models

	Model 1		Model 2¶		Model 3††	
	Crude OR (95% CI)	*P*	Adjusted OR (95% CI)	*P*	Adjusted OR (95% CI)	*P*
Stable normal-calcium	Ref.	-	Ref.	-	Ref.	-
Very low TSC	7.0 (3.8–13.0)	< 0.001	8.4 (4.4–15.8)	< 0.001	7.2 (3.7–14.0)	< 0.001
Moderately low TSC	5.3 (4.0-6.9)	< 0.001	5.3 (4.0–7.0)	< 0.001	5.0 (3.8–6.7)	< 0.001
Fluctuating high TSC	4.8 (1.4–16.6)	0.014	5.4 (1.5–19.2)	0.009	5.6 (1.5–20.6)	< 0.01

TSC, total serum calcium; OR, odds ratio; CI, confidential interval

¶Adjusted for age, gender, race

††Adjusted for age, gender, race, congestive heart failure, chronic pulmonary disease, chronic renal failure, metastatic cancer, and liver disease

## Discussion

We reported a novel approach to identify sub-phenotypes in patients with AP on the basis of TSC trajectories. Through group-based trajectory modeling, we identified four groups of patients that follow distinct TSC trajectories. We found significant physiological differences between the trajectory groups, as well as considerable variation in in-hospital mortality, with the “Very low TSC, slow resolvers” having a mortality rate six times higher than the “Stable normal-calcium”. We found that patients with fluctuating high level TSC also showed a higher mortality. These findings have important significance for understanding the heterogeneity of AP and may inform future research to determine treatment strategy across different sub-phenotypes.

Recent evidence proved that the identification of biologically meaningful subgroups of a disease could lead to more personalized management strategies. For example, Bhavani and colleagues identified sub-phenotypes with different inflammatory responses in sepsis using temperature trajectories [23], with the sub-phenotypes displaying different clinical outcomes. Similarly, Bhatraju and colleagues [24] identified sub-phenotypes of acute kidney injury (AKI) based on the creatinine trajectory, with the finding that non-resolving patients had 68% higher mortality. AP is a late entry to the field of sub-phenotyping. Neyton and colleagues [25] used unsupervised clustering of transcriptomic, proteomic, and metabolomic data to describe four sub-phenotypes of AP (have not yet been subject to peer review; www.researchgate.net/publication/330852956). Although the results are promising, the findings are based on small sample size (54 cases) and it costly and complicated to use in practice. The simple, routine, and widespread laboratory test of serum calcium have great potential for AP phenotyping, because it is readily available, inexpensive, and have standardized reference ranges. The universal availability of longitudinal TSC trajectories could facilitate more resource-intensive diagnostics for the classification of clinically relevant AP sub-phenotypes.

It is well documented that point-specific hypocalcemia is significantly associated with AP severity, and hypocalcemia is one of the components of Ranson’s criteria to assess the severity of AP [16, 26, 27]. Peng and colleagues have recently reported that serum calcium on admission is independently associated with persistent organ failure in AP and may serve as a potential prognostic factor [28]. Notably, we discovered that transient hypocalcemia (TSC < 8.4 mg/dL) was more common in non-survivors compared with survivors (70.3% vs. 30.6%) in the 24 h from presentation. We also reported that TSC variability was higher in non-survivors than in survivors. Our study underscores the value of evaluating the trajectory of the TSC rather than a static test; and these data support the prognostic implication of dynamic TSC measurements and their benefit over using a single TSC measurement.

Importantly, we discovered four TSC trajectory groups: “Very low TSC, slow resolvers”; “Moderately low TSC”; “Stable normal-calcium”; and “Fluctuating high TSC”. Studies have elucidated the mechanisms of extracellular calcium entry and calcium-mediated acinar cell injury and death in AP [19], so we hypothesize that the four groups might represent the different degree and duration of pancreatic parenchymal injury. Given the low mortality rate of the “Stable normal-calcium”, we assume that they may represent a transient calcium influx and quickly recovered sub-phenotype. For “Very low TSC, slow resolvers” and “Moderately low TSC” phenotypes, they may represent a relatively longer duration of calcium toxicity induced organelle dysfunction sub-phenotypes. For the three groups, serum calcium should be interpreted as the mediator of gallstones, alcohol, drugs or any other causes that trigger the pathological cellular pathways and organelle dysfunction in AP. For “Fluctuating high TSC” group, we hypothesize that hypercalcemia secondary to calcium infusion, malignancy (e.g., some from osteoclastic damage from metastatic cancer to bone), hyperparathyroidism or other disease might be the direct cause of AP.

Identifying the specific phenotypes could lead to a better understanding of the pathophysiology of AP and may inform new treatment targets. It’s well known that the mitochondrial dysfunction secondary to cellular calcium overload leads to ATP depletion, and ultimately results in acinar cell necrosis [29, 30]. Based on the central importance of calcium concentration toxicity, ORAI1 channel inhibitors that prevent extracellular calcium enter into the acinar cells have been developed [31, 32]. ORAI1 inhibitors have been demonstrated to prevent necrosis in mouse models of AP and human acinar cells, alleviating both the local and systemic extent of organ injury [31]. Furthermore, TRO40303 was found to prevent ATP depletion caused by cytosolic calcium overload, which also has therapeutic potential for AP [33]. The benefit of these novel strategies that targeting calcium overload in AP should be validated in future multicenter trial. This has shed light on the new treatment approach of AP, especially in severe AP that still has a high mortality rate up to now.

Our study has some limitations that should be acknowledged. First, The MIMIC database consists of data collected from electronic health records from 2001 to 2019; this has led to significant missing data for inflammatory markers, such as ESR and CRP. Thus, we couldn’t confirm the differential inflammatory basis for these hypothesized sub-phenotypes. In addition, the management of AP may have changed over this period of time, which might bring confounding effects on our analysis. Third, we did not control for the etiology of AP due to its unavailable in the database, and further study are needed to confirm whether these sub-phenotypes are consistent in AP caused by different etiology. Fourth, we could not remove the effect of calcium infusion on TSC trajectories. However, modelling based on AP patients who received calcium infusion within the first 24 h didn’t change the TSC trajectories in a significant way compared with patients without calcium infusion (Figs. S1 and S2). Furthermore, the TSC concentration might be influenced by the level of serum albumin. However, sensitivity analysis that modelling based on the serum ionized calcium (Fig. S3) showed similar trajectories, demonstrating that the phenotyping was stable.

## Conclusions

In conclusion, we have discovered four novel TSC trajectory sub-phenotypes of AP, and this approach provides a framework for prognosticating distinct sub-phenotypes of patients with AP. Our results suggest that the four groups may represent differential mode and extent of pancreatic injury. If confirmed to relate to the underlying calcium toxicity, TSC trajectory groups could identify treatment responsive sub-phenotypes and lead to improved personalization of patient management.

### Electronic supplementary material

Below is the link to the electronic supplementary material.

Supplementary Material 1

## Data Availability

All data generated or analyzed during this study are included in this published article.

## Abbreviations

AP: Acute pancreatitis
AKI: Acute kidney injury
BUN: Blood urea nitrogen
CRP: C-reactive protein
IQR: Interquark range
RRT: Renal replace treatment
TSC: Total serum calcium (TSC)
