# Supplementary material for: Identifying novel acute pancreatitis sub-phenotypes using total serum calcium trajectories
Source: BMC Gastroenterol. 2024 Apr 23;24:141. doi: 10.1186/s12876-024-03224-9 (PMC11036611; doi:10.1186/s12876-024-03224-9)
Supplement: Supplementary file 1 — Supplementary Material 1 [file 12876_2024_3224_MOESM1_ESM.docx]

**Table S1** Comparison of baseline characteristics between survivors and non-survivors

|  | Non-survivors  (n = 259) | survivors  (n = 4259) | P |
| --- | --- | --- | --- |
| Age (years) | 70.1 ± 35.8 | 56.9 ± 24.5 | <0.001 |
| Male, n (%) | 142 (54.8) | 2217 (52.1) | 0.422 |
| Race, n (%) |  |  | <0.001 |
| White | 160 (61.8) | 2918 (68.5) |  |
| Black | 16 (6.2) | 578 (13.6) |  |
| Other | 83 (32.0) | 763 (17.9) |  |
| Comorbidities, n (%) |  |  |  |
| Congestive heart failure | 78 (30.1) | 431 (10.1) | <0.001 |
| Chronic pulmonary disease | 48 (18.5) | 690 (16.2) | 0.369 |
| Chronic renal failure | 47 (18.1) | 492 (11.6) | 0.002 |
| Metastatic cancer | 20 (7.7) | 106 (2.5) | <0.001 |
| Liver disease | 92 (35.5) | 800 (18.8) | <0.001 |
| Disease severity and laboratory indexes |  |  |  |
| SOFA score (median [IQR]) | 9.0 [6.0, 13.0] | 4.0 [2.0, 7.0] | <0.001 |
| Minimum albumin, mg/dL | 2.7 ± 0.6 | 3.3 ± 0.7 | <0.001 |
| Minimum bicarbonate, mmol/L | 18.8 ± 6.2 | 23.5 ± 4.6 | <0.001 |
| Maximum bilirubin, mg/dL | 4.8 ± 7.3 | 1.8 ± 3.1 | <0.001 |
| Maximum creatinine, mg/dL | 2.3 ± 2.0 | 1.3 ± 1.5 | <0.001 |
| Maximum BUN, mg/dL | 42.0 ± 28.7 | 19.1 ± 18.5 | <0.001 |
| Maximum glucose, mg/dL | 192.1 ± 124.0 | 137.2 ± 87.9 | <0.001 |
| Maximum white blood cell count (10^9/L) | 16.5 ± 10.9 | 10.8 ± 7.3 | <0.001 |
| Maximum amylase, IU/L | 185.5 [69.2, 658.0] | 155.0 [65.2, 494.5] | 0.222 |
| Maximum lipase, IU/L | 246.0 [55.0, 1148.0] | 172.0 [52.0, 749.0] | 0.06 |
| Initial TSC, mg/dL | 7.8 ± 1.2 | 8.5 ± 0.9 | <0.001 |
| Maximum TSC, mg/dL | 8.2 ± 1.1 | 8.6 ± 0.8 | <0.001 |
| Minimum TSC, mg/dL | 7.5 ± 1.2 | 8.4 ± 0.9 | <0.001 |
| Mean TSC, mg/dL | 7.9 ± 1.0 | 8.5 ± 0.8 | <0.001 |
| TSC, SD | 0.5 ± 0.4 | 0.3 ± 0.3 | <0.001 |
| Time to trough TSC, h | 10.8 ± 6.8 | 12.3 ± 5.9 | <0.001 |
| Hypocalcaemia (TSC<8.4 mg/dL), n (%) | 182 (70.3) | 1644 (38.6) | <0.001 |
| Clinical outcomes |  |  |  |
| In-hospital death |  |  |  |
| ICU admission, n (%) | 241 (93.1) | 1413 (33.2) | <0.001 |
| Hospital length of stay, days, (median [IQR]) | 13.1 [4.1, 25.2] | 5.1 [3.0, 9.9] | <0.001 |
| Antibiotic use, n (%) | 218 (84.2) | 1933 (45.4) | <0.001 |
| Intubation, n (%) | 168 (64.9) | 457 (10.7) | <0.001 |
| RRT, n (%) | 70 (27.0) | 73 (1.7) | <0.001 |

SOFA, Sequential Organ Failure Assessment; BUN, Blood urea nitrogen, TSC, total serum calcium; SD, standard deviation; RRT, renal replace treatment

**Table S2** Selection of different group-based temperature-trajectory phenotypes

| Group-based trajectory model | BIC | AvePP | | | |
| --- | --- | --- | --- | --- | --- |
|  |  | Phenotype-1 | Phenotype-2 | Phenotype-3 | Phenotype-4 |
| One trajectory group | −21332.27 | 1.0 ± 0.0  (n = 5204) |  |  |  |
| Two trajectory groups | −19333.28 | 0.89 ± 0.15 (n = 819) | 0.95± 0.09  (n = 4385) |  |  |
| Three trajectory groups | −18246.88 | 0.90 ± 0.15 (n = 784) | 0.96 ± 0.08 (n = 4383) | 0.96 ± 0.11 (n = 37) |  |
| Four trajectory groups | −17417.56 | 0.92 ± 0.13  (n = 65) | 0.85 ± 0.16  (n = 559) | 0.90 ± 0.13 (n = 3875) | 0.96 ± 0.10 (n = 19) |

Abbreviations: BIC, Bayesian information criterion; AvePP, average posterior probability


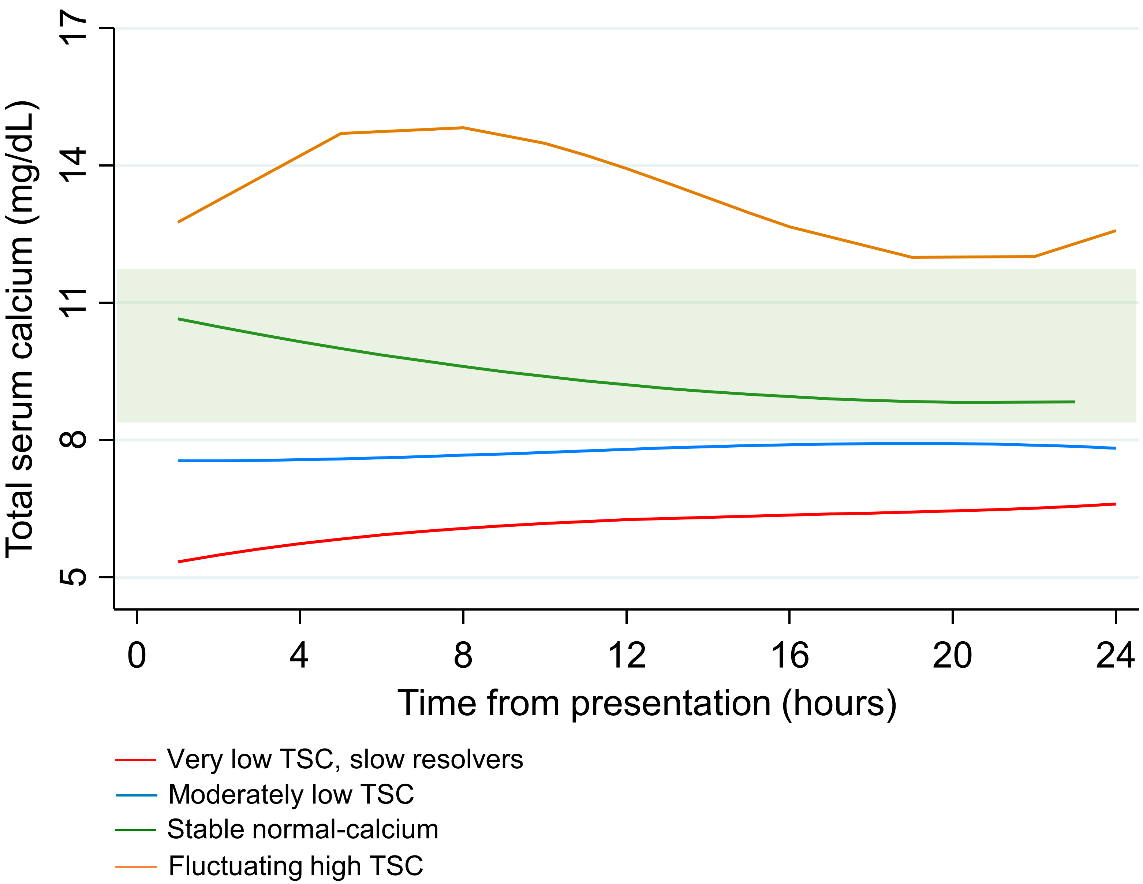


**Fig. S1** Four TSC trajectory phenotypes in acute pancreatitis (AP) patients who received calcium infusion within the first 24 h after hospital admission (n=358).


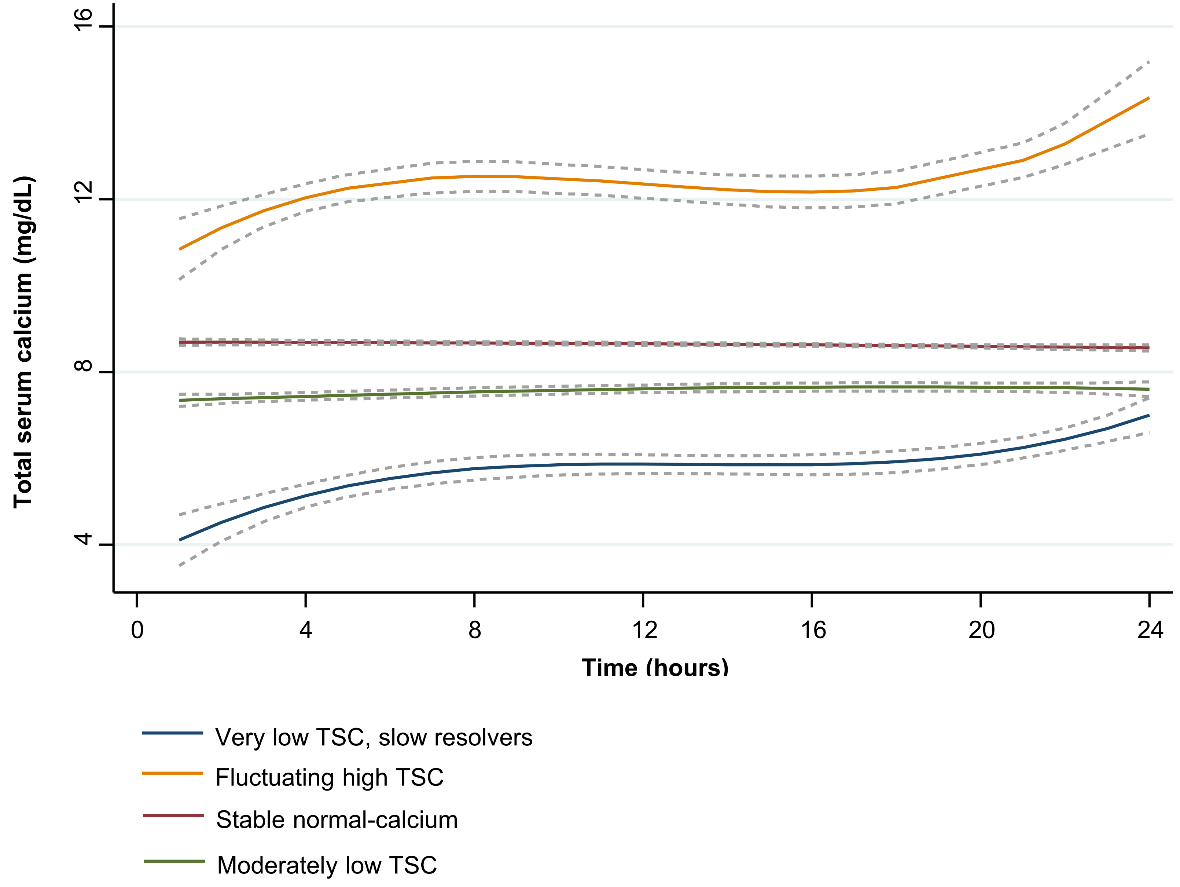


**Fig. S2** Four TSC trajectory phenotypes in acute pancreatitis (AP) patients who didn’t receive calcium infusion within the first 24 h after hospital admission (n=4160).

**
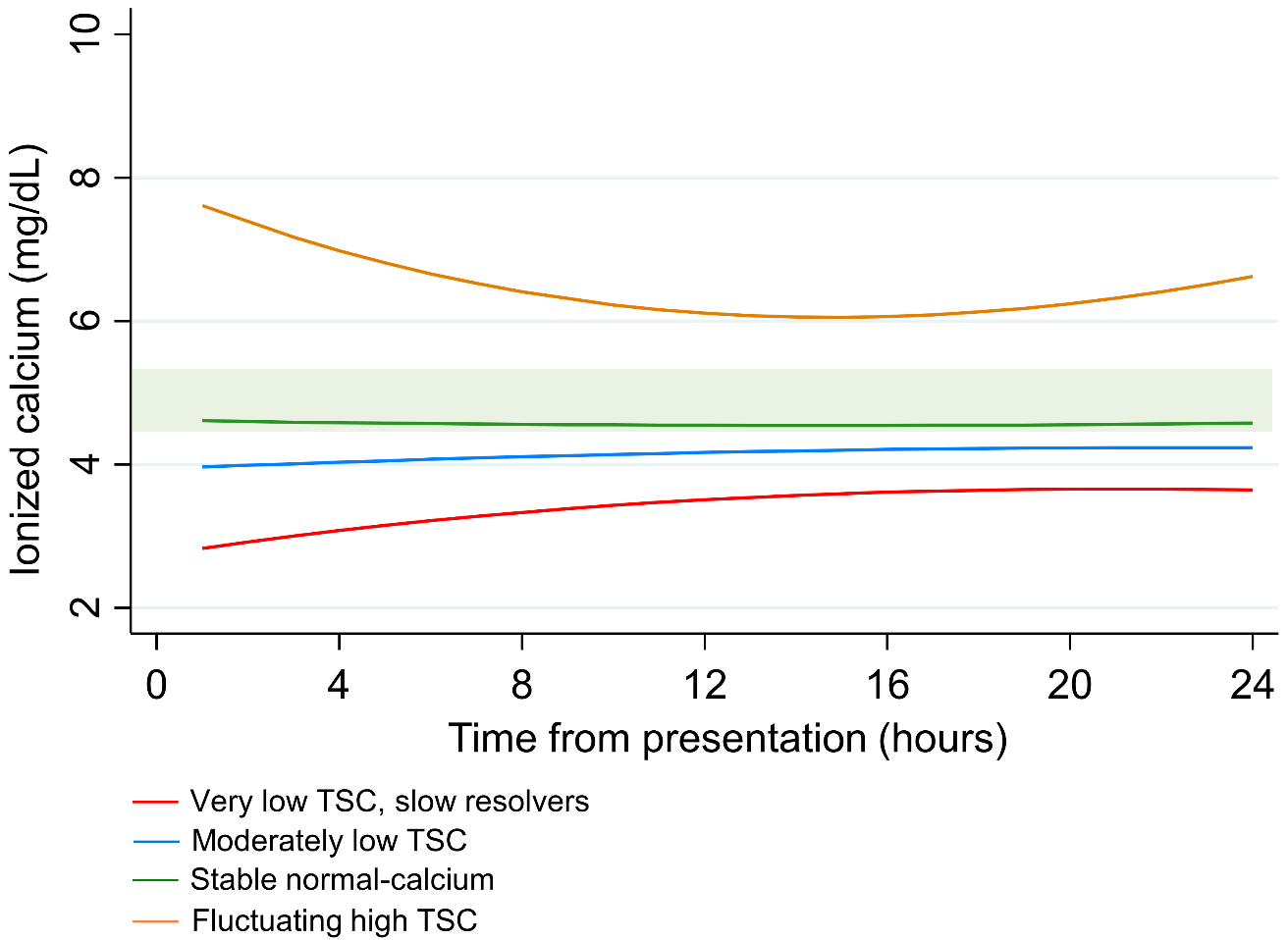
Fig. S3** Four TSC trajectory phenotypes in acute pancreatitis (AP) patients based on available ionized calcium data (n=923).
